# Lipedema in a male patient: report of a rare case – management and review of the literature

**DOI:** 10.3205/iprs000161

**Published:** 2021-09-22

**Authors:** Mattis Bertlich, Mark Jakob, Ines Bertlich, Reggy Schift, Randolf Bertlich

**Affiliations:** 1 Department of Otorhinolaryngology, Head and Neck Surgery, Ludwig-Maximilians-University, Munich, Germany; 2 Dermafit Institute for Aesthetic Dermatology, Marl, Germany; 3 Department of Dermatology, University Hospital, University of Heidelberg, Heidelberg, Germany; 4 Dermatologic Practice Reggy Schift, Amstelveen, The Netherlands

**Keywords:** lipedema, liposuction, tumescence liposuction

## Abstract

**Objective:** Lipedema is a relatively common yet debilitating and often misdiagnosed lipodystrophy that mainly affects females. Very little is known about the etiology and pathophysiology of the disease. However, due to its high preference for female patients, hormonal factors may contribute to the pathogenesis.

**Case:** A 62-year-old male patient presented to the authors with painful swelling of the thighs. The patient had been treated elsewhere for lymphedema with subsequent disease progression. Lipedema stage IV was confirmed by clinical examination and ultrasound. The patient underwent three sessions of tumescence liposuction which was well tolerated. Later on, the patient reported great improvement in terms of complaints as well as disfigurement.

**Conclusion:** The etiology and pathophysiology of lipedema remain unclear. However, the case at hand shows that lipedema may, albeit rare, also present in male patients. Moreover, we show that liposuction is efficient and safe in treating lipedema even in atypical cases.

## Zusammenfassung

**Hintergrund:** Das Lipödem ist eine häufige, jedoch auch stark beeinträchtigende und häufig fehldiagnostizierte Lipodystrophie, die in erster Linie Frauen betrifft. Ätiologie und Pathophysiologie sind unbekannt. Aufgrund der starken Präferenz für das weibliche Geschlecht werden hormonelle Ursachen immer wieder in Betracht gezogen.

**Fall:** Ein 62 Jahre alter, männlicher Patient stellte sich den Autoren aufgrund einer schmerzhaften Schwellung der Oberschenkel vor. Zuvor war auswärtig die Diagnose eines Lymphödems gestellt und dieses mit Lymphdrainagen behandelt worden. Klinisch zeigte sich ein Lipödem °IV. Das Lipödem wurde durch dreimalige Tumeszenzliposuktion behandelt. Im Anschluss kam es zu einer relevanten Besserung der Beschwerden.

**Schlussfolgerung:** Ätiologie und Pathophysiologie des Lipödems bleiben unklar. Anhand des vorliegenden Falles wurde gezeigt, dass Lipödeme, obwohl im Vergleich mit der relevanten Literatur ausgesprochen selten, auch Männer betreffen können. Darüber hinaus zeigt der vorliegende Fall, dass auch untypische Lipödeme sicher und effektiv mit Liposuktionen behandelt werden können.

## Introduction

Lipedema is a disease that is defined by unphysiological accumulation of fat in the upper and lower extremities. Extension is commonly from the shoulders to the wrist and from the hip to the ankles, actually sparing the hands and feet, making it an often relatively straightforward clinical diagnosis that needs to be differentiated from obesity and lymphedema. However, it is still often misdiagnosed [1]. Unlike obesity, the unphysiological accumulation of subcutaneous fat does not respond to dietary changes or exercise [2]. In addition to the mentioned considerable deformity, patients suffering from lipedema experience considerable pain in the affected areas [3]. During the further course of the disease, patients regularly experience increased tendency to bruising and secondary lymphedema [3]. Finally, a great many patients suffering from lipedema eventually develop psychologic disorders [4]. So overall, albeit not life threatening, lipedema may severely affect quality of life in patients [5].

The exact causes of lipedema remain unclear to this day. There are some hereditary conditions like the Williams syndrome that are associated with lipedema [6]. Moreover, there are some families where an autosomal-dominant hereditary pattern has been observed [7]. Nonetheless, these hereditary cases are of a more anecdotic nature; in most patients lipedema is considered to be idiopathic. There is, however, one more feature of lipedema that is striking: Apart from the syndromal cases, it almost exclusively arises in females. 8 to 17 percent of all adult women may eventually be affected from the disease [1]. This gives rise to the hypothesis that there may also be an hormonal etiology to lipedema.

Treatment is dependent on the stage of the disease and ranges from conservative treatment options like compression treatment in the early stages of the disease to surgical options like liposuction in more advanced stages.

However, there are still many aspects of lipedema that are not well understood to this day. In the report at hand we present a rare case of a male patient that presented with lipedema.

## Case description

A 62-year-old patient presented to the authors of this study in late 2016 with an increase in circumference of both thighs. The increase had started without any specific trigger in the past year, was slowly progressing and limited to the thighs and, although significantly less extensive, the calves. In terms of general diseases, the patient had suffered from type I diabetes for decades and was treated for arterial hypertension. Regular medication included ibesartan, carvedilol and nifedipine. Body mass index was 31.4 kg/m^2^.

Before presentation to the authors of this study, the patients had been treated elsewhere and a diagnosis of lymphedema had been made. Subsequent treatment of manual lymphatic drainage had been applied twice a week for six months, without any sign of improvement. Upon presentation, the patient complained about the deformity of his legs which had become debilitating by itself. Not only did the patient’s clothes fit no longer, the inner sides of the thighs had come in contact during walking, causing constant irritation. Additionally, the patient complained from increased bruising and considerable touch dependent pain of the thighs.

**Figure 1:**
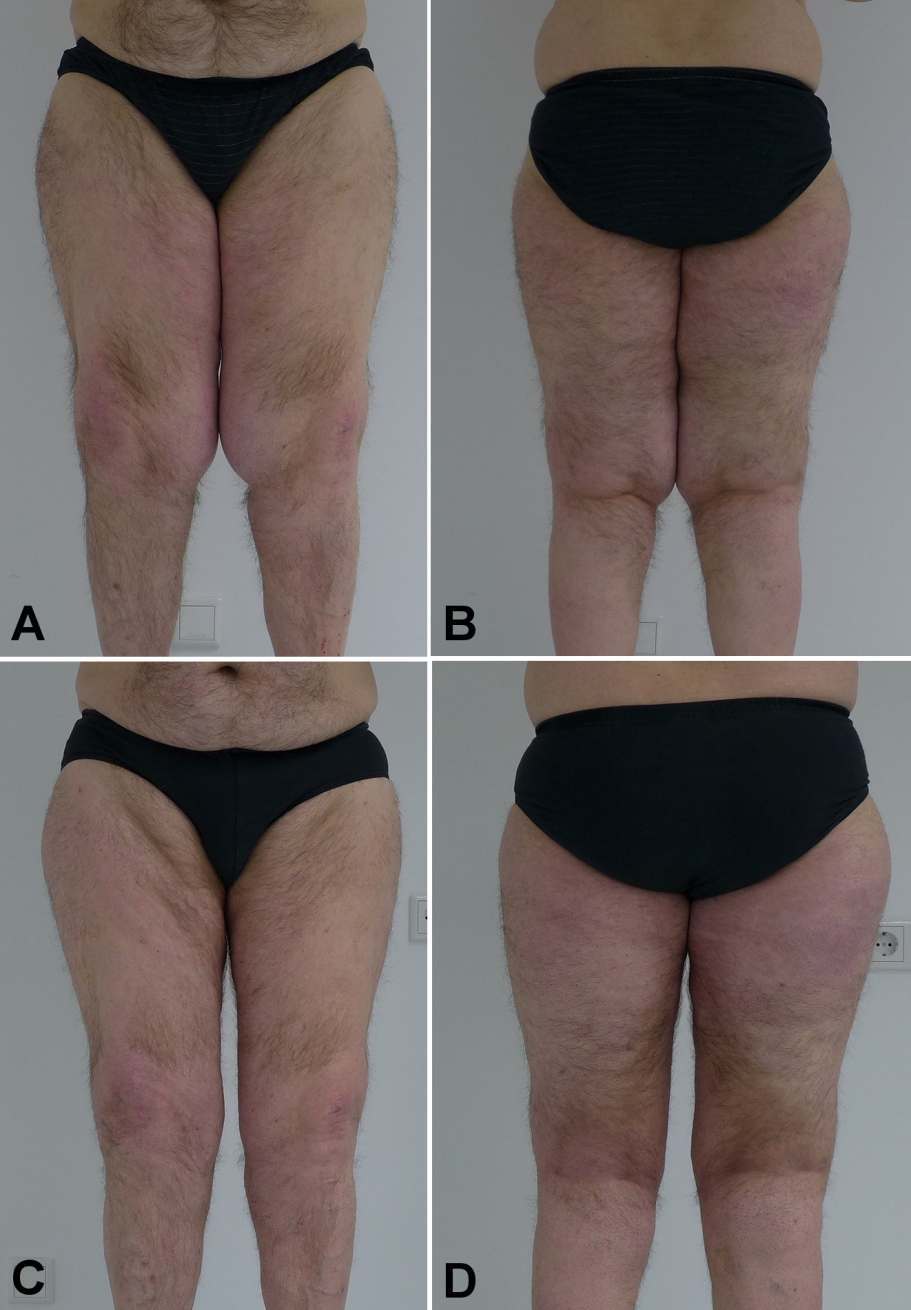
A/B – Patients lower extremity front and back views before tumescence liposuction was performed, B/C – lower extremity front and back four months after the first set of liposuctions.

Diagnosis of stage II lipedema with subsequent lipolymphedema was confirmed by clinical inspection (Figure I A/B (Fig. 1)) as well as sonography of the thighs and calves. Bloodwork including hemostaseology, electrolyte, inflammation markers and hormones was, apart from a slightly elevated serum glucose and cholesterol, unremarkable. After explaining diagnosis and treatment options to the patients, the decision for surgical intervention was taken. After consultation with the patients insurance, permission for tumescence liposuction was given.

**Figure 2:**
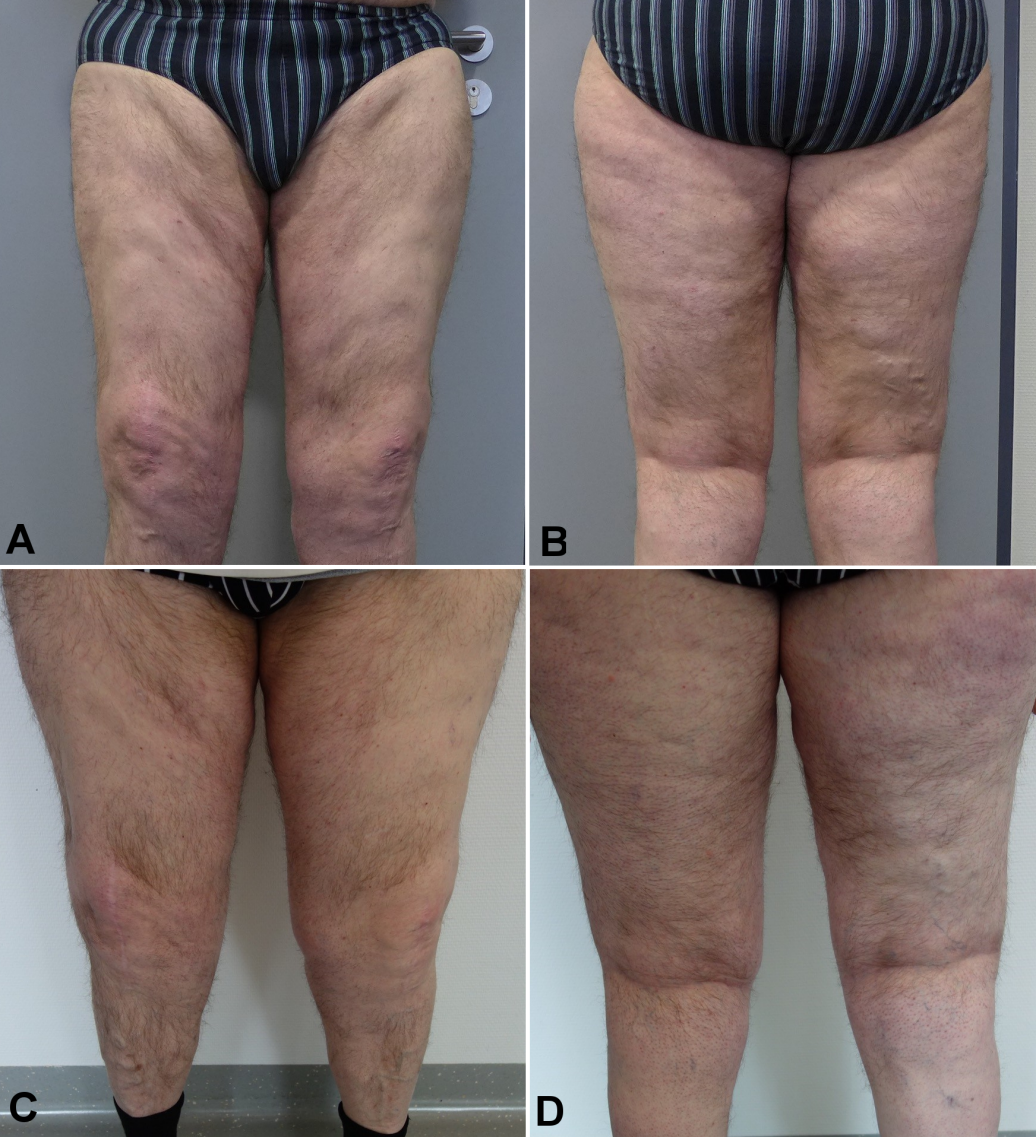
A/B lower extremities two months after the third session of liposuction, C/D lower extremities 2.5 years after liposuction. Note that the patient had gained 20 kg of additional bodyweight.

The patient underwent three sessions of tumescence liposuction from November 2016 to August 2017. Overall, a little over 8,000 ml of fat were removed (2,100 ml, 4,400 ml and 2,500 ml of fat for each session, respectively). Liposuction was well tolerated and caused a significant decrease in thigh volume and circumference (Figure 1 C/D (Fig. 1) and Figure 2 A/B (Fig. 2)). Histological samples showed no malignancy, consistent with the diagnosis of lipedema. The patient subsequently reported a great improvement of the complaints and returns for regular check-ups on a yearly basis. To this date, the patient has remained free of lipedema recurrence (Figure 2 C/D (Fig. 2)).

## Discussion

Lipedema is a relatively common [3], often misdiagnosed [1], [8] and also hard to treat [9] disease that almost exclusively affects females. Most of the literature, including the German S1-guideline in treating lipedema [10], is commonly limited to stating that lipedema “mainly affects women”. However, after a thorough literature research, we were only able to identify cases where lipedema occurring in males was associated with a syndromal disease6 as well as two cases where idiopathic lipedema occurred in a male patient [11], [12]. Hence, we are able to present – to the best of our knowledge – a very rare case of idiopathic male lipedema. This observation raises two important issues: Firstly, differential diagnosis of lipedema needs to be considered in male just as well as female patients. Moreover, it gives insight into the potential etiology of lipedema in male patients: Compared to the other two cases that have been reported in scientific literature, it is remarkable that hormonal abnormalities have been observed in two of those patients. While one patient in literature had previously suffered from ethyltoxic liver cirrhosis [11], the patient at hand had a history of alcohol abuse as well as type I diabetes. Taking into account that lipedema almost exclusively affects females, we believe that the role of the hormones in the pathophysiology of the disease can hardly be underestimated.

The second important aspect of the case highlights the need of a timely diagnosis: Since lipedema is often initially misdiagnosed [1], [8], [9] and correct diagnosis subsequently delayed, we believe that this may be the case particularly in lipedema in males. This assumption is underlined by the fact that the patient at hand was initially treated elsewhere for lymphedema for several months and was repeatedly referred until he eventually presented with considerable symptoms to the authors of this study.

Moreover, the case at hands highlights the viewpoint that tumescence liposuction is both safe and effective in treating lipedema – even so in rare or unusual cases. There has been quite some debate – particularly in Germany – as to whether liposuction in lipedema should be covered by the general insurance [13], [14]. However, there is substantial evidence that liposuction is effective and lasting in treating lipedema [2], [3], [10], [15], [16], [17], which is in line with the case at hand.

Finally, the case at hand as well as the literature available on this topic highlight that lipedema is, although relatively common, very poorly understood, particularly its etiology and pathophysiology. However, treatment has been and is both straightforward and effective, with conservative regiments in early stages and liposuction for advanced stages and patients refractory after conservative therapy.

## Conclusion

We reported a rare case of idiopathic male lipedema. This case highlights that idiopathic lipedema not only occurs in females and needs to be considered as a differential diagnosis in male patients as well. Moreover, liposuction is a safe and efficient mean of treating advanced lipedema, even in atypical cases.

## Notes

### Competing interests

The authors declare that they have no competing interests.
